# STR Profiling for Discrimination between Wild and Domestic Swine Specimens and between Main Breeds of Domestic Pigs Reared in Belarus

**DOI:** 10.1371/journal.pone.0166563

**Published:** 2016-11-16

**Authors:** Krzysztof Rębała, Alina A. Rabtsava, Svetlana A. Kotova, Viachaslau N. Kipen, Natalja V. Zhurina, Alla I. Gandzha, Iosif S. Tsybovsky

**Affiliations:** 1 Department of Forensic Medicine, Medical University of Gdansk, Gdansk, Poland; 2 Scientific and Practical Centre of the State Committee of Forensic Expertises, Minsk, Belarus; 3 Scientific and Practical Centre of the National Academy of Sciences on Animal Husbandry, Zhodino, Belarus; China Agricultural University, CHINA

## Abstract

A panel comprising 16 short tandem repeats (STRs) and a gender-specific amelogenin marker was worked out and tested for robustness in discrimination between wild and domestic swine subspecies encountered in Europe, between regional populations of wild boars and between main breeds of domestic pigs reared in Belarus. The STR dataset comprised 310 wild boars, inhabiting all administrative regions of Belarus, and 313 domestic pigs, representing three local and three cosmopolitan lines. Additionally, a total of 835 wild boars were genotyped for the presence of melanocortin 1 receptor (*MC1R*) alleles specific for domestic pigs. Correctness of assignment of STR profiles to appropriate populations was measured by log-likelihood ratios (log-LRs). All samples were correctly identified as wild boars or domestic pigs with average log-LR of 42.4 (LR = 2.6×10^18^). On the other hand, as many as 50 out of 835 (6.0%) genotyped wild boars from Belarus possessed *MC1R* alleles specific to domestic pigs, demonstrating supremacy of our STR profiling system over traditional differentiation between wild boars and domestic pigs, based on single binary markers. Mean log-LRs for allocation of wild boars to their regions of origin and of domestic pigs to appropriate breeds were 2.3 (LR = 9.7) and 13.4 (LR = 6.6×10^5^), respectively. Our results demonstrate the developed STR profiling system to be a highly efficient tool for differentiation between wild and domestic swine subspecies and between diverse breeds of domestic pigs as well as for verification of genetic identity of porcine specimens for the purpose of forensic investigations of wildlife crimes, assurance of veterinary public health, parentage control in animal husbandry, food safety management and traceability of livestock products.

## Introduction

Discrimination between wild and farmed species of animals is of basic importance for assessment of food authenticity, its safety management, assurance of veterinary public health and forensic investigations of wildlife crimes [1,2], and usefulness of molecular genetics in this field is unquestionable [3–5]. One of challenges encountered in such studies concerns two closely related subspecies: European wild boars (*Sus scrofa scrofa*) and domestic pigs (*Sus scrofa domesticus*) [6]. In Belarus, an Eastern European state with more than 9.4 million hectares of forests and with 39% forestation, wild boars used to be the most common game mammals, hunted both legally and illegally, with tens of thousands of individuals shot legitimately per year and an unknown number of poaching cases. Thus, tons of wild boar meat were officially introduced to the market, carrying temptation of fraudulent substitution with cheaper domestic pork and the need for reliable speciation and traceability of meat products. Furthermore, court proceedings of cases of illegal hunting often require species identification and individualisation of biological samples in order to provide indisputable evidence of a crime. Apart from simple species detection, individualisation and/or kinship analysis of porcine samples is also essential for parentage control in animal husbandry and for investigation of thefts of livestock or meat products. Moreover, the need for animal identification and traceability has been further boosted by the recent outbreak of African swine fever in Eastern Europe and the need to control the spread of the epidemic [7].

The aim of the present study was to work out a method based on DNA analysis by polymerase chain reaction (PCR) and on statistical inference of genotypes by likelihood ratios (LRs) for simple discrimination between wild boars and domestic pigs as well as for individualisation of the tested porcine samples and their assignment to regional wild populations or to distinct domestic breeds reared in Belarus. For the purpose of our study, a panel of 17 DNA markers was worked out, which comprised 16 microsatellites (short tandem repeats, STRs) and amelogenin locus for gender detection.

## Materials and Methods

**Fig 1:**
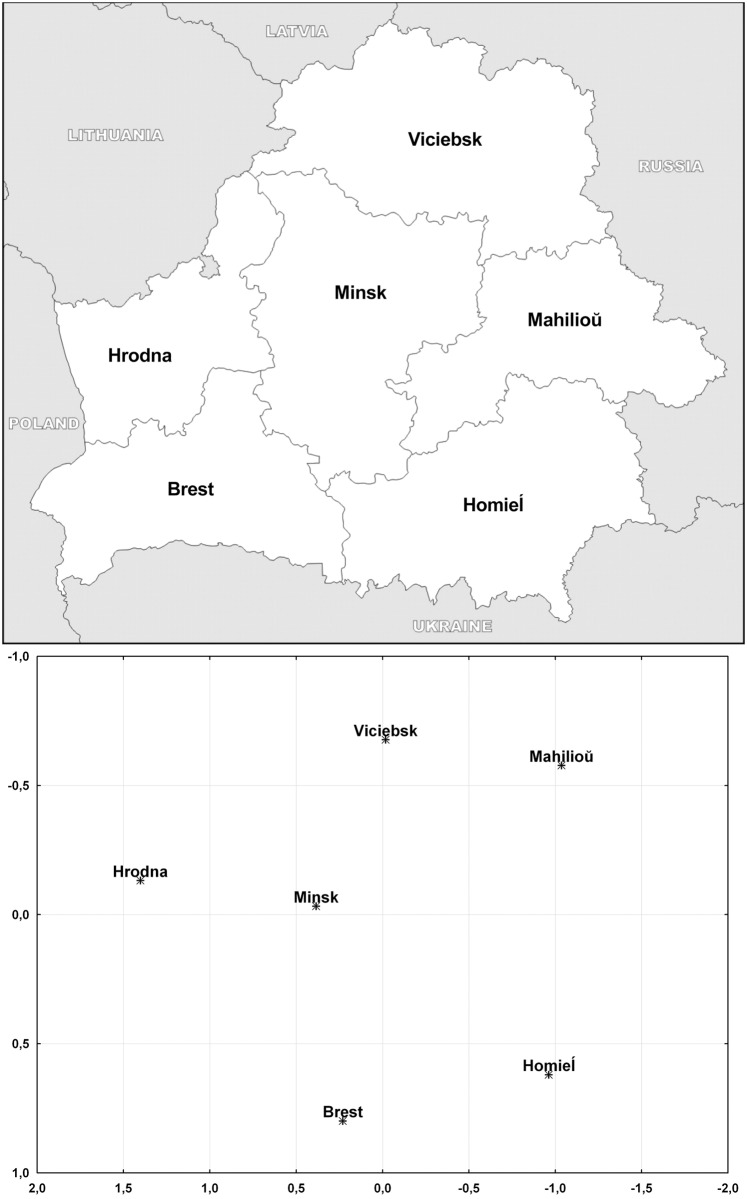
Geographic location of 6 administrative regions of Belarus, in which wild boar samples were collected (adapted from http://www.d-maps.com/carte.php?num_car=4238&lang=en), compared with a two-dimensional plot obtained from MDS based on pairwise F_ST_ values for 6 regional wild boar populations from Belarus and for 16 STR markers analysed in the study.

In the first phase of the study, a total of 835 samples (fragments of ears or muscles) were collected from European wild boars hunted legally in all administrative regions of Belarus (Fig 1): Brest (n = 176), Viciebsk (n = 149), Homieĺ (n = 85), Hrodna (n = 152), Minsk (n = 121) and Mahilioŭ (n = 152). The animals were not killed for research purposes and the hunters had all appropriate permits. Simultaneously, samples were obtained from 129 domestic pigs selected randomly throughout the country regardless of the represented breed and were provided by slaughterhouses operating within state-owned farms: “Zachodni” (Brest region), “Ahrakambinat Snoŭ” (Minsk region), “Zadniaproŭski” (Viciebsk region) and “Zarečča” (Homieĺ region). In the second phase, a total of 304 domestic pigs representing 6 most common breeds were enrolled into the study, including 3 local breeds: Belarusian Meat (n = 53), Belarusian Large White (n = 50) and Belarusian Black Pied (n = 20), as well as 3 breeds reared in many countries, including Belarus: Landrace (n = 54), Yorkshire (n = 55) and Duroc (n = 72). Nine individuals from the first phase of the study remained unassigned in the second phase and represented Piétrain breed and interbreed hybrids.

For DNA extraction, muscle and cartilage tissue samples were incubated with occasional shaking at 37–56°C in lysis buffer containing 2% sodium dodecyl sulfate (SDS), 20 mM Tris-HCl pH 8.0, 100 mM NaCl, 20 mM EDTA and proteinase K. Lysates of boar tissues were purified with the use of silica gel, whereas lysates obtained from tissues of domestic pigs underwent purification with phenol and chloroform in the presence of 1.0 M sodium perchlorate (NaClO_4_), followed by precipitation with 1 volume of isopropyl alcohol. The dried DNA pellets were dissolved in TE buffer consisting of 10 mM Tris-HCl pH 8.0 and 0.1 mM EDTA.

All wild boars were genotyped by analysis of restriction fragment length polymorphism (RFLP) of melanocortin 1 receptor (*MC1R*) gene for the presence of alleles specific for domestic pigs according to a method developed by Fajardo et al. [8]. Genotyping of STR markers was performed with the use of three multiplex PCRs (Table 1). All domestic pigs, all wild boars carrying the domesticated *MC1R* allele (n = 50) and 260 individuals selected from the subgroup of wild boars without the *MC1R* allele specific for domestic pigs underwent STR profiling. The wild boars included in STR analysis represented the regions of Brest (n = 65), Viciebsk (n = 61), Homieĺ (n = 30), Hrodna (n = 44), Minsk (n = 52) and Mahilioŭ (n = 58). For all multiplex systems, amplification was carried out in an iCycler thermocycler (Bio-Rad) in a final volume of 10 μl containing 200 mM KCl, 7.5 mM MgCl_2_, 0.2 mM dNTPs, 0.08–8 ng DNA and 0.75 U *Taq* polymerase. The reactions consisted of initial denaturation (3 min 45 s at 95°C), 36 cycles of amplification (45 s at 95°C, 45 s at 60°C, 1 min 30 s at 72°C) and terminal elongation (30 min at 72°C). PCR products were separated electrophoretically in a 3500 Genetic Analyzer (Applied Biosystems). Alleles of the tested loci were identified by size in base pairs (bp) with the use of GeneMapper ID-X software by comparison to a GeneScan 600 LIZ v2.0 internal size standard.

**Table 1:** Characterisation of DNA markers used in the study.

Marker	STR size	Chromosome	Primer sequences	Fluorescent label	Multiplex	No. of alleles	Allele range	Reference
FH1589	4	3	F:CACAGGAGCAGCCCTAGATAA R:AGGAATTGGGTAGAAGTTCGTG	ROX	3	21	136–204	[9]
FH1696	4	1	F:AGAGGCTTCTCACTAAACTCTGG R:ACTTTGCTGAATTCCTTGTTCG	TAMRA	2	15	345–397	[9]
FH1701	4	5	F:CTTGTCAGCCTGTAGCAAATATC R:AAGGAAACCAATAGTATGGAGATG	FAM	2	15	175–231	[9]
FH1727	4	15	F:TCGCTATGACATTTCTAAACAGAT R:TTACTGCCAGGGTTTCTCAAAT	R6G	1	13	206–254	[9]
FH1733	4	11	F:AAGCCTCAAACTCCTCATCTCA R:ACCAAAGGCATACTAGGGCTAA	ROX	1	10	274–314	[9]
FH1900	4	1	F:GCAATGACCAACATGCAAA R:TGAAAGGAGCACTGAGCTTACA	FAM	2	10	231–279	[9]
FH2148	4	1	F:TCTGGTTCTGTCCCTAGCC R:GGGCTTCTCTCTCCTCCTACA	FAM	3	21	236–376	[9]
FH2478	4	12	F:ACTGAAGCAGCTCGGGTCAC R:GAGGGAAGTTGAGGGTCTTATTC	R6G	2	10	272–308	[9]
FH2709	4	2	F:AGCCACCAGAGAACCCTAAATA R:GGTACGGGCCTAAGAAACA	R6G	2	20	114–186	[9]
FH3637	4	11	F:AGGAAACTGAATGCCCTCTCTC R:CTGGCTTGGGAATTTGCAT	FAM	1	17	133–323	[9]
NLRIP0001	4	17	F:GATCTCAGCTTCAATACCTCC R:GATCCTGTATTGCTGTGGCTG	R6G	1	13	333–381	[10]
S0005	2	5	F:TCCTTCCCTCCTGGTAACTA R:GCACTTCCTGATTCTGGGTA	ROX	3	32	193–269	[11]
S0101	2	7	F:GAATGCAAAGAGTTCAGTGTAGG R:GTCTCCCTCACACTTACCGCAG	ROX	2	13	193–219	[11]
S0766	4, 2	6	F:GTGTAGATATGTGTCTGTACA R:AGACCTCCTATTAGAGGTGGA	FAM	1	9	434–464	[12]
SW240	2	2	F:AGAAATTAGTGCCTCAAATTGG R:AAACCATTAAGTCCCTAGCAAA	R6G	1	18	93–127	[13]
SW857	2	14	F:TGAGAGGTCAGTTACAGAAGACC R:GATCCTCCTCCAAATCCCAT	ROX	2	11	136–160	[13]
Amelogenin	–	X, Y	F:GTTTAAGCCCTGATGGGTCA R:CCGGGATAGAACTCTGGTCA	FAM	3	2	171–181	[14]

Allele frequencies, observed and expected heterozygosity values as well as P values testing for Hardy-Weinberg equilibrium (HWE) were obtained with the use of Arlequin 3.1 software [15]. A non-parametric Wilcoxon signed-rank test was carried out using STATISTICA 12 software (StatSoft) to test for differences in observed and expected heterozygosity. Matching probability, defined as probability that two genotypes sampled at random from a population will be identical, was calculated with the aid of PowerStats 1.2 [16]. Interpopulation differentiation of the tested STRs was assessed in Arlequin 3.1 by analysis of molecular variance (AMOVA). Linearised pairwise F_ST_ values were applied in multidimensional scaling (MDS) analysis with the use of STATISTICA 12. The same software was employed to conduct genotype assignment for pairs of populations. In order to estimate a minimal number of markers required to discriminate both swine subspecies, the analysis was carried out with the use of the whole panel of 16 STRs and the same panel with sequential elimination of the least informative loci (as assessed by F_ST_ values obtained in locus-by-locus AMOVA). The obtained log-likelihoods of individual genotypes coming from populations of wild boars and domestic pigs were used to obtain log-likelihood ratios (log-LRs) of an individual representing wild boar vs. domestic pig population. The performance of log-LRs for different numbers of markers were subsequently evaluated in STATISTACA 12 by receiver operating characteristic (ROC) curves, for which area under curve (AUC) was computed.

## Results

As many as 50 wild boar individuals (6.0%) showed mixed RFLP patterns with *MC1R* alleles specific to both wild boars and domestic pigs. Frequency of the allele specific to domestic pigs in the Belarusian wild boars was estimated to reach 3.0%. Percentage of wild boars with the domesticated allele varied across regions from 4.1% in Minsk to 8.6% in Mahilioŭ, but AMOVA did not reveal regional stratification of its occurrence within the country (F_ST_ = –0.00155; P = 0.71287). Distribution of *MC1R* genotypes followed Hardy-Weinberg equilibrium in the general wild boar population and all its regional subgroups (P = 1.00000). AMOVA of 16 tested STR markers did not detect any traces of genetic differentiation between unadmixed and admixed individuals from the Belarusian wild boar population (F_ST_ = –0.00207; P = 0.99475). Thus, in all subsequent analyses, both groups were treated as one wild boar population.

Allele frequencies observed in both swine subspecies samples (*Sus scrofa scrofa* and *Sus scrofa domesticus*) and in the different domestic pig breeds for the tested 16 STRs are presented in S1 Table. Statistically significant genetic differentiation between both swine subspecies was shown in AMOVA (F_ST_ = 0.08577; P<0.00001). Locus-by-locus analysis revealed that all the tested markers contributed to the observed genetic differentiation with F_ST_ values extending from 0.01487 up to 0.16005 for FH3637 and FH1900 loci, respectively. Corresponding P values for all the markers did not exceed 0.00001 with the exception of the least differentiating one, FH3637, with P = 0.00188 (Table 2).

**Table 2:** Impact of the tested STR markers sorted in an ascending order of F_ST_ values on discrimination between wild boars and domestic pigs.

Marker	F_ST_^a^	P^a^	AUC for log-LRs^b^	Average log-LR for assignment to the correct population^b^	Minimal log-LR for assignment to the correct population^b^	Percentage of incorrect assignments for log-LR cut-off point equal to 0^b^
FH3637	0.01487	0.00188	1.0000	42.4	6.8	0.0%
FH1589	0.02975	0.00000	1.0000	41.4	7.2	0.0%
FH1696	0.04626	0.00000	1.0000	40.3	5.8	0.0%
FH2478	0.06049	0.00000	1.0000	38.8	7.8	0.0%
FH1701	0.06066	0.00000	1.0000	37.3	6.3	0.0%
FH1727	0.07341	0.00000	1.0000	35.6	3.2	0.0%
SW240	0.07645	0.00000	1.0000	33.4	4.0	0.0%
S0005	0.08687	0.00000	1.0000	30.7	2.2	0.0%
FH2709	0.08715	0.00000	1.0000	23.6	1.0	0.0%
NLRIP0001	0.08759	0.00000	1.0000	18.5	-1.1	0.9%
FH2148	0.10062	0.00000	0.9998	15.1	-2.7	1.4%
S0766	0.10779	0.00000	0.9992	11.6	-2.6	1.8%
FH1733	0.11438	0.00000	0.9966	9.2	-3.1	3.6%
S0101	0.12005	0.00000	0.9918	7.7	-4.5	4.3%
SW857	0.14338	0.00000	0.9621	4.3	-6.2	10.7%
FH1900	0.16005	0.00000	0.8878	2.0	-4.5	17.3%

^*a*^ locus-by-locus AMOVA between wild boar and domestic pig populations from Belarus

^*b*^ before sequential elimination of the marker from the first column

Analysis of impact of the tested STR loci on discrimination of wild boars and domestic pigs revealed that as few as 7 most informative markers were sufficient to correctly assign all genotyped individuals to the proper subspecies, based on the optimal log-LR cut-off point determined by means of the ROC curve, as shown by AUC values in Table 2. However, the computed optimal log-LR cut-off points differed from 0 and therefore, from the statistical point of view, automatically supported one of two tested hypotheses. Thus, we additionally calculated the number of incorrect assignments for log-LR cut-off points equal to 0 and found out that LRs for 7 markers indicated wrong populations in case of 4 individuals (0.9%) and that 8 was the minimal number of the most informative loci required to accurately separate wild and domestic swine subspecies. As inferred from minimal log-LR values, even for 16 markers, there was an individual with log-LR of only 6.8 for correct assignment (Table 2), corresponding to LR equal to 8.8×10^2^. However, this was an extreme case, as far as mean log-LR reached 23.6 (LR = 1.7×10^10^) for 8 most informative STRs and 42.4 (LR = 2.6×10^18^) for the whole panel of 16 markers.

The number of detected alleles, values of observed and expected heterozygosity as well as P values testing for Hardy-Weinberg equilibrium for each of the tested markers in each of the studied swine populations are collected in S2 Table. Wilcoxon test revealed statistically significant differences in observed heterozygosity values between populations of wild boars and domestic pigs (P = 0.008) and for all but one of comparisons of randomly selected domestic pigs and their distinct breeds (P<0.05). In general, wild boars and all domestic pig breeds displayed statistically higher observed heterozygosity values than the sample of domestic pigs collected randomly throughout the country apart from Duroc breed, whose observed heterozygosity values were statistically lower than those of randomly selected domestic pigs. The only breed which showed insignificant difference in observed heterozygosity values in comparison to the random pig sample was Belarusian Black Pied, but its sample numbered only 20 individuals. Regarding expected heterozygosity, wild boars from the Homieĺ region were found to have statistically lower values than the general wild boar population (P = 0.049). Also representatives of Duroc breed showed statistically lower expected heterozygosity values in comparison to the random domestic pigs from Belarus (P = 0.0004), but this parameter for this breed was significantly reduced in relation to all the other studied domestic pig breeds (P<0.01). Furthermore, observed heterozygosity was statistically lower than expected one in the general wild boar population, in wild boars from the Brest and Mahilioŭ regions and especially in the random domestic pigs from Belarus, whose mean expected heterozygosity amounted to 0.766 and was comparable to its value in all the other studied population samples (except for Duroc breed), but was much higher than mean observed heterozygosity in this population, reaching only 0.664 (Table 3).

**Table 3:** Comparison of the mean number of detected alleles, mean observed (Ho) and expected (He) heterozygosity as well as the number of statistically significant deviations from HWE at the tested loci in the studied swine populations.

	No. of genotyped individuals	Mean no. of detected alleles per locus	Mean Ho per locus	Mean He per locus	No. of significant HWE deviations	No. of significant HWE deviations after Bonferroni correction
Wild boars	310	11.8	0.772	0.799	9	5
Brest	65	9.7	0.762	0.797	6	2
Viciebsk	61	9.4	0.776	0.799	6	2
Homieĺ	30	8.4	0.790	0.785	7	0
Hrodna	44	8.7	0.764	0.790	10	2
Minsk	52	9.3	0.798	0.801	5	0
Mahilioŭ	58	8.8	0.753	0.790	7	3
Domestic pigs^a^	129	10.6	0.664	0.766	15	11
Belarus Meat	53	8.8	0.764	0.773	1	0
Belarus Large White	50	8.8	0.765	0.783	1	0
Belarus Black Pied	20	6.8	0.713	0.735	0	0
Landrace	54	8.8	0.765	0.757	2	0
Yorkshire	55	7.9	0.725	0.735	1	1
Duroc	72	6.8	0.603	0.595	5	4

^*a*^ randomly selected throughout the country regardless of the represented breed

A total of 9 and 15 loci revealed deviation from HWE (P<0.05) in the wild boar and random domestic pig populations, respectively. After application of Bonferroni correction, the number of HWE departures decreased to 5 in wild boars and 11 in domestic pigs. Matching probability for loci in HWE after Bonferroni adjustment reached 1 in 3.7×10^12^ and 1 in 7.6×10^3^ in wild and domestic swine from Belarus, respectively. There were 5–10 markers in Hardy-Weinberg disequilibrium in regional Belarusian wild boar populations (P<0.05). The correction for multiple testing limited statistical significance to 2–3 loci in 4 regional populations and removed it totally in 2 regional populations. Drastic reduction of the number of STRs deviating from HWE was observed when distinct breeds of domestic pigs from Belarus were analysed separately. There were 0–2 markers showing HWE departure (P<0.05) in the studied breeds excluding Duroc and none but one deviation from HWE in these breeds after Bonferroni correction. Less apparent decrease in the number of loci in Hardy-Weinberg disequilibrium was noted in representatives of Duroc breed with 5 and 4 markers departing from HWE before and after correction for multiple testing, respectively (Table 3).

AMOVA of regional wild boar populations demonstrated geographic stratification of the tested STR markers within the country (F_ST_ = 0.00641; P<0.00001). All pairwise comparisons between regional populations were statistically significant (P<0.05) with the smallest F_ST_ distances (P>0.01) observed for the centrally located population of the Minsk region and between neighbouring populations of southern Belarus (Homieĺ and Brest) and eastern Belarus (Homieĺ and Mahilioŭ; S3 Table). Genetic distances between regional wild boar populations visualised by MDS almost perfectly reflected their geographic positions (Fig 1). For the whole panel of 16 STRs, mean log-LRs for assignment of 310 wild boars to their region of origin vs. random wild boars was 2.3 (LR = 9.7×10^0^). For the log-LR cut-off point equal to 0, as many as 38 individuals (12.3%) were less likely to come from an appropriate region rather than represent a random wild boar from Belarus.

**Fig 2:**
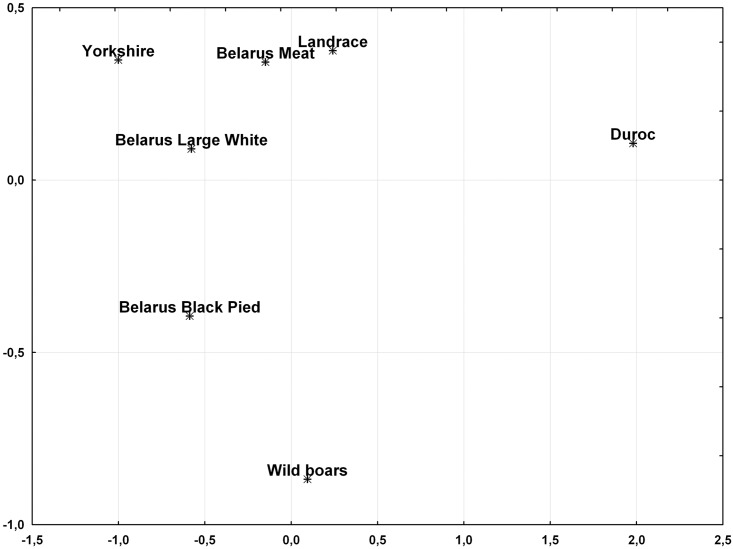
A two-dimensional plot obtained from MDS based on pairwise F_ST_ values for 6 distinct domestic pig breeds from Belarus compared with the general wild boar population and for 16 STR markers analysed in the study.

Much more profound genetic differences were found in case of pairwise F_ST_ values obtained for the studied domestic pig breeds with P<0.00001 for all pairwise comparisons. The smallest F_ST_ value was obtained in comparison of Belarusian Meat and Landrace pigs (S3 Table) and was also visible in the MDS plot (Fig 2). On the other hand, the most profound genetic distances were observed between Duroc breed and other swine populations, including wild boars, and were approximately twofold larger than genetic distances between all the other breeds (S3 Table). Genetic distinctiveness of the Duroc individuals was also confirmed in the first dimension of the MDS plot, in which wild boars grouped together with other studied domestic pig breeds and departed from the rest only in the second dimension (Fig 2). For the whole panel of 16 STRs, mean log-LRs for assignment of 304 domestic pigs representing 6 most common breeds to the proper breed vs. random domestic pigs was 13.4 (LR = 6.6×10^5^). For the log-LR cut-off point equal to 0, only 6 individuals (2.0%) were less likely to come from the appropriate breed rather than from random domestic pigs from Belarus. In case of 9 individuals unassigned to any breed in the second phase of the study (3 Piétrain pigs and 6 interbreed hybrids), the maximal log-LR values of an individual representing one of six breeds vs. random domestic pigs were negative in 6 cases (66.7%) and reached maximally 5.1 (LR = 1.6×10^2^) for assignment of a Belarusian Large White × Piétrain hybrid to Belarusian Large White breed.

## Discussion

Species identification of biological samples, including discrimination between specimens of wild and farmed animals, has paid a lot of attention of agricultural and food scientists, veterinarians and forensic practitioners, and a number of techniques of DNA analysis have been developed for this purpose. One of the best established methods applied in species identification and phylogenetic studies is based on sequencing of mitochondrial genes encoding cytochrome b (*cyt b*) and cytochrome oxidase I (*COI*) [17]. However, *cyt b* sequencing failed to distinguish between wild boars and domestic pigs due to high homology of their mitochondrial sequences [18]. Discovery of biallelic markers with genetic variants specific to only one swine subspecies opened possibilities for their discrimination and was originally based on PCR-RFLP analysis of mitochondrial D-loop region [19] and of nuclear genes such as melanocortin 1 receptor (*MC1R*) [8] or nuclear receptor subfamily 6, group A, member 1 (*NR6A1*) gene [20]. Furthermore, a number of other candidate genes with very different minor allele frequencies in wild and domestic swine were identified by recent whole-genome sequencing studies [21]. However, usefulness of the D-loop region in swine subspecies identification has been questioned due to maternal inheritance of mitochondrial DNA, restricting correct identification of hybrids [8]. Also single biallelic nuclear markers have been shown to possess limited potential in swine subspecies discrimination due to introgression of domesticated alleles to wild boars, which was detected in populations of Southern, Central and Western Europe [20,22–25] and was confirmed in our Eastern European wild boar population from Belarus with *MC1R* heterozygotes constituting 6.0% of the genotyped individuals. Combined analysis of several biallelic polymorphisms was shown to significantly improve genetic discrimination between wild boars and domestic pigs; however, it does not completely eliminate the risk of incorrect assignments [20].

For this reasons, attention of researchers has shifted towards panels of multiple markers, which usually enable unequivocal differentiation of wild and domestic *Sus scrofa* subspecies and in addition offer opportunity of individualisation of the tested biological samples. A notable example of such a panel is a commercially available PorcineSNP60 BeadChip (Illumina), a genome-wide high-density single nucleotide polymorphism (SNP) genotyping array, which detects over 64,000 evenly distributed porcine SNPs and was shown to exhibit a powerful potential for identification of wild boars, domestic pigs and their hybrids [23,26]. Another markers which have found application in swine subspecies detection are multiallelic microsatellites, which are routinely analysed by multiplex PCR technique, whose extreme sensitivity is highly desirable in forensic casework and currently overcomes the one of Illumina porcine SNP genotyping assays. Since the principal question in legal investigations of poaching and food deception is about wildlife or livestock origin of the tested porcine samples, our assignment of an individual to a proper swine subspecies with the use of multiallelic STR markers with differing allele frequencies employs a probabilistic Bayesian approach, in which LRs support and provide quantitative evidence for one of two predefined alternative hypotheses about the origin of the tested sample [27]. In order to implement this approach in forensic casework in Belarus, we have worked out STR allele frequency databases for randomly sampled wild boars and randomly sampled domestic pigs, representative for the country as a whole, which provide valuable data for reliable population assignment in practice.

Several panels of porcine STR markers have been developed so far, which were used in parentage control in breeding farms [28,29] as well as in investigation of crimes involving poaching and cruelty to a wild animal [30] and veterinary malpractice [31]. A commercially available Animaltype Pig multiplex PCR test kit (Biotype) comprising 11 STR markers has been used by Caratti et al. for development of an allele frequency database for wild boars and domestic pigs from northwestern Italy for statistical assessment of DNA evidence in practice [32]. The authors applied Bayesian cluster analysis to correctly assign 97.4% of wild boars and 99.1% of domestic pigs to relevant populations. Another DNA identification system was developed by Lin et al. and was tested on domestic pig breeds and Formosan wild boars (*Sus scrofa taivanus*), but its robustness in discrimination of both subspecies was not assessed [33]. Conyers et al. worked out a panel of 20 porcine microsatellites for differentiation of European wild boars from domestic pig breeds in food with 100% correct assignment to appropriate groups [34]. On the contrary to the Animaltype Pig test kit investigated by Caratti et al., our log-likelihoods obtained in the genotype assignment test based on allele frequencies estimated in each population sample allowed for correct assignment of all the tested individuals to wild boar or domestic pig populations with the use of as few as 8 most informative markers. However, legal investigations require not only indication of a more likely hypothesis, but also its statistical evaluation, usually in the form of LR. Our results demonstrate that the minimal log-LR value observed in our dataset for the correct population assignment increases from 1.0 for 8 markers to 6.8 for the whole panel of 16 STRs, corresponding to LRs of 3 and 8.8×10^2^, respectively. In forensic casework, DNA evidence is interpreted as extremely strong when LR reaches 10^6^ (log-LR = 13.8) [27], and this value is actually easily attainable for our panel of 8–16 microsatellites for discrimination between wild boars and domestic pigs, as one can conclude from the mean log-LR values shown in Table 2. Thus, our results demonstrate exceptional usefulness of the developed panel of porcine STRs for differentiation of the two studied swine subspecies.

Investigation of crimes related to poaching or theft of livestock as well as identification and traceability of individuals in herds and in biodiversity conservation programmes often require individualisation of biological samples rather than simple species detection. In such cases, statistical assessment of DNA evidence consists in estimation of probability of observing identical STR profiles in a given population and demonstration of its uniqueness limited to the tested sample. The rarer a genetic profile in the population, the stronger evidence that matching profiles come from the same individual. Probability of observing an identical DNA profile requires evaluation of its frequency, based on frequencies of STR alleles in the population. However, allele frequencies may be directly employed for estimation of frequency of a genetic profile only when distribution of alleles in the population is consistent with Hardy-Weinberg law [35]. Our population samples of wild boars and domestic pigs randomly selected throughout the country displayed statistically significant differences in allele frequencies at all the tested loci and departure from HWE after correction for multiple testing for 5 and 11 markers, respectively. Although exclusion of 5 STRs from the panel of 16 markers for estimation of genetic profile frequency in a wild boar population should not affect considerably evidence value of DNA typing, restriction of statistical assessment of DNA evidence to 5 loci exhibiting HWE out of 16 tested markers in domestic pigs provides matching probability insufficient for definite evidence. However, separate analysis of distinct breeds of domestic pigs practically removed significant deviations from HWE, explaining the observed Hardy-Weinberg disequilibrium and statistically significant excess of homozygotes in randomly selected domestic pigs from Belarus as Wahlund effect, caused by population subdivision [35].

Apart from swine subspecies detection, DNA markers have found application in identification of distinct breeds of domestic pigs for the purpose of traceability and authentication of livestock products. Alves et al. analysed porcine mitochondrial DNA and identified SNPs specific to Iberian and Duroc pigs [36]. Another SNP in a nuclear gene encoding v-kit Hardy-Zuckerman 4 feline sarcoma viral oncogene homolog (*KIT*) was found to be useful for authentication of meat products obtained from Cinta Senese pigs reared exclusively in Italy [37]. A 96-plex SNP genotyping assay using markers selected from the PorcineSNP60 BeadChip was shown to be a powerful tool for identification of traditional and commercial breeds of domestic pigs from the United Kingdom [38]. Likewise, microsatellite markers were used to distinguish between Berkshire and Jeju Black pigs in Korea [39]. Profound differences in STR allele frequencies between the most common domestic pig lines from Belarus and our results of genotype assignment tests imply usefulness of the developed panel of STR markers in identification of the studied domestic pig breeds. Genetic separation of Duroc breed from other breeds and from European wild boars, as revealed in pairwise comparisons and MDS, may result from intense selection by breeders during the past decades, leading to a reduced effective population size, as far as observed and expected heterozygosity averaged over 16 loci was much lower than in the other groups. However, one cannot neglect a fact that Duroc pigs originated in America unlike Landrace, Yorkshire and Belarusian local lines which were developed in Europe. Genetic distinctiveness of this breed was also observed by Megens et al., who studied microsatellite variation in 98 pig lines from Europe and China and found Duroc to be the most divergent among breeds developed in Europe or derived from European pigs [40]. Genetic proximity between Landrace and Belarusian Meat breeds, revealed in our analysis, was not surprising, taking into account Landrace ancestors of Belarusian Meat pigs [41].

In general, when population substructure is observed, separate allele frequency databases should be developed for each genetically distinct subpopulation and used in practice for evaluation of DNA evidence. So far, regional variation of STR markers in Belarus has been well characterised only in local human populations [42]. Our results of AMOVA revealed statistically significant regional stratification in Belarusian wild boar populations, which seems to be moderate in comparison to genetic differences observed between domestic pig breeds from Belarus. Moreover, pairwise F_ST_ distances and results of MDS suggest this substructure is strongly driven by geography. Significant genetic differences in STR allele distribution between regional European wild boar populations, as observed in our study, provide potential for assignment of genotyped individuals to their geographic origin for the purpose of tracking illegal trade [43]. However, obtained evidence values measured by log-LRs for assignment of Belarusian wild boars to their regions of origin were low and generally insufficient for unambiguous inference. Nevertheless, one should take into account that the compared local wild boar populations were defined by administrative rather than geographic boundaries and inhabit a relatively small area in Europe with fairly unrestricted gene flow. The geography-driven genetic differences between regional wild boar populations, revealed in Belarus, are likely to grow together with the geographic distance within the continent, allowing for clear differentiation e.g. between wild boars from Eastern and Western Europe.

In summary, the proposed panel of 16 porcine STR markers constitutes an effective tool for discrimination between wild and domestic swine subspecies encountered in Europe and between distinct domestic pig breeds, as well as for verification of genetic identity for the purpose of forensic investigations of wildlife crimes, assurance of veterinary public health, parentage control in animal husbandry, food safety management and traceability of livestock products.

## Supporting Information

S1 Table Allele frequencies of 16 STR markers in the studied population samples of wild boars and domestic pigs from Belarus. (XLS)

S2 Table Number of detected alleles, observed and expected heterozygosity as well as P values testing for HWE in the studied population samples of wild boars and domestic pigs from Belarus. (XLS)

S3 Table Pairwise F_ST_ values and corresponding P values for AMOVA of 16 STR markers in wild boar populations from 6 administrative regions of Belarus and in domestic pig populations from Belarus, representing 6 distinct breeds, compared to the general Belarusian wild boar population. (XLS)
